# Glucocorticoid receptor mutations and clinical sensitivity to glucocorticoid in Chinese multiple sclerosis patients

**DOI:** 10.1007/s10072-020-04376-8

**Published:** 2020-04-10

**Authors:** Tian Song, Haoxiao Chang, Li Du, Linlin Yin, Fudong Shi, Xinghu Zhang

**Affiliations:** 1 Department of Neurology, Beijing Tiantan Hospital, Capital Medical University, Beijing, 100070 China; 2 China National Clinical Research Center for Neurological Diseases, Beijing, 100070 China

**Keywords:** Glucocorticoid receptor, Mutations, GRα, FKBP5, Multiple sclerosis

## Abstract

**Background:**

Glucocorticoid (GC) is the first-line therapy in acute attacks of multiple sclerosis (MS), but its efficacy is individually variable and may be associated with glucocorticoid receptor (*GR*) gene.

**Objective:**

To establish the association between *GR* gene sequence and clinical GC sensitivity in Chinese MS patients. And to investigate the expression differences of serum GRα and FK506 binding protein 5 (FKBP5) in GC responders and non-responders.

**Materials and methods:**

Coding exons 2–9 of the *GR* gene from 97 MS patients were sequenced. We performed ELISA to detect serum GRα and FKBP5 before the GC impulse therapy in patients with different GC sensitivities (according to the EDSS changes before and after the GC medication).

**Results:**

Seven new mutations were located in exon 2, but the presence or absence of mutations was not associated with the response to GC therapy (*P* = 0.416). The GC-sensitive patients had higher GRα (*P* = 0.011) but lower FKBP5 (*P* = 0.025) levels in the serum.

**Conclusions:**

The *GR* mutations detected in our study were not associated with the response to GC in Chinese MS patients. Higher GRα and lower FKBP5 levels in the serum might predict the response to GC, which may provide potential therapeutic target for GC-resistant patients with acute MS attack.

**Electronic supplementary material:**

The online version of this article (10.1007/s10072-020-04376-8) contains supplementary material, which is available to authorized users.

## Introduction

Multiple sclerosis (MS) is an inflammatory disease of central nervous system (CNS) which generally begins in early adulthood. Glucocorticoids (GC) have anti-inflammatory and immunosuppressive properties and are thus recommended as the first-line therapy in the management of acute attacks of MS [1]. However, in the clinical practice, we found individual variability in GC efficacy. Some patients were resistant to GC initially, but in some other patients, the response to GC attenuated with relapses (secondly resistant) [2]. In recent years, several large-scale databases reveal this point in real-world observational studies [3].

The mechanism governing the responsiveness of GC action remains elusive [4, 5]. Multiple factors can influence cellular glucocorticoid sensitivity at the level of the glucocorticoid receptor (GR) and its signaling pathway, including co-chaperones such as FK506-binding protein 5 (FKBP5) [6, 7]. The *GR* gene can give rise to multiple splice variants: GRα (the most abundant isoform), GRβ, GRγ. GRα is a transcription factor with transcriptional regulatory activity and essential to GC sensitivity regulation [8]. Researches in Caucasians report that polymorphisms of *GR* result in a modified transcript, which may have an impact on GC sensitivity and disease course [9–11]. However, no research on Chinese MS patients has been conducted so far.

In this study, we detected *GR* gene alterations in the entire coding regions in Chinese MS patients and aimed to elucidate the association of *GR* gene and the GC response. We also investigated the differences in GRα and FKBP5 concentrations in GC responders and non-responders to explore possible strategies for modulation of glucocorticoid reactivity.

## Methods

### Patients

A total number of 97 Chinese patients fulfilling the revised 2017 McDonald criteria for MS or clinical isolated syndrome (CIS) were included in this study [12]. Of them, 38 patients were diagnosed with CIS, whereas 44 with relapsing-remitting MS (RRMS), 12 with secondary progressive MS (SPMS), and 3 with primary progressive MS (PPMS). All patients were in the acute attack and received glucocorticoid impulse therapy (methylprednisolone 500 mg/days for 5 days). The Expanded Disability Severity Scale (EDSS) was assessed before the glucocorticoid impulse and 7 days after the therapy. Sixty-two patients responded to GC (the differences of EDSS before and after the GC impulse ≥ 0.5 [13]), whereas 35 patients were non-responders (no differences in EDSS before and after the GC impulse [13]). The clinical data and characteristics of the patients are listed in Table 1.

**Table 1:** Demographic and clinical characteristics of 97 MS (CIS) patients

	CIS *n* = 38	RRMS *n* = 44	SPMS *n* = 12	PPMS *n* = 3
Gender(male/female)	17/21	18/26	3/9	1/2
Age (year, M ± SD)	23.1 ± 7.5	32.9 ± 6.7	44.2 ± 10.3	37.7 ± 8.4
EDSS before GC (M ± SD)	1.6 ± 0.7	2.8 ± 1.3	3.3 ± 0.8	3.5 ± 1
EDSS after GC (M ± SD)	1.3 ± 0.5	2.5 ± 1.1	3.1 ± 0.6	3.5 ± 1

This study was approved by the Ethics Committee of Beijing Tiantan Hospital, Capital Medical University, and written informed consent was obtained from all enrolled individuals.

### DNA extraction and sequence analysis

Peripheral blood samples were taken on the day before GC impulse during active stage of disease. Genomic DNA was isolated from peripheral blood mononuclear cell (PBMC) using a whole-blood genomic DNA extraction kit (BioTeke, Beijing). The coding exons 2–9 of the *GR* gene (NM_001018077) were PCR-amplified by a LifeECO gene amplification instrument (BIOER, UK). The PCR products were bidirectionally sequenced employing an ABI 3730XL Genetic Analyzer (Life Technologies, USA). The primers used in the investigation are listed in the supplementary materials.

### ELISA assay

GRα and FKBP5 levels were analyzed by ELISA in 20 serum samples in the GC-sensitive group and 18 in the resistant group. The levels of GRα and FKBP5 were measured on the same day before the GC treatment. Assays were performed following the instructions of the kit (Elabscience, E-EL-H1998c, CHN for GRα/AVIVA, OKEH00685, USA for FKBP5). Data were presented as the average of duplicate results.

### Statistical analysis

Differences in the mutation distribution among GC responders and non-responders were analyzed by Fisher’s exact test. Data were analyzed using independent sample *t* test for normally distributed data (concentration of GRα) and the Mann-Whitney test for non-normally distributed data (concentration of FKBP5). All statistical analysis was performed by IBM SPSS statistical software version 21.0 (IBM, Armonk, NY, USA). Values of *P* < 0.05 were considered statistically significant.

## Results

Whole-exome sequencing of the GR gene revealed 56 gene alterations, including 7 new unreported mutations and 49 single-nucleotide polymorphisms (SNPs), which are common in East Asian populations. All these seven mutations were located in exon 2 in the transcriptional activation region (TAR) of the GR gene, including five missense mutations and two premature termination codons (PTC) (Table 2). Six mutations were heterozygous and one mutation was homozygous. These seven mutations can cause changes in the coding amino acids and were predicted to be damaging or probably damaging by SIFT and Polyphen 2 software. Interestingly, unrelated patients No. 4 and No. 5 had an identical A > T missense mutation on chromosomal chr5:142779668.

**Table 2:** The seven mutations and patients’ data

Patient	Gender	Age	Diagnosis	EDSS change	Chromosomal location	Nucleotide change	Amino acid change
1	Male	31	CIS	1	chr5:142780172	c.233T>C	p.L78P
2	Female	42	RRMS	1.5	chr5:142779906	c.499C>T#	p.Q167*
3	Male	70	CIS	0	chr5:142779543	c.862A>G	p.T288A
4	Female	38	RRMS	2	chr5:142779668	c.737A>T	p.K246M
5	Male	26	CIS	1.5	chr5:142779668	c.737A>T	p.K246M
6	Female	49	CIS	1	chr5:142779822	c.583C>T	p.Q195*
7	Female	64	SPMS	0.5	chr5:142780253	c.152C>T	p.A51V

# indicate homozygous genotype

The mutation distributions in the MS patients based on their response to GCs (responders and non-responders) are presented in Table 3. No significant difference in the mutation distribution was detected between the responders and non-responders to GC therapy (*P* = 0.416).

**Table 3:** Mutation distributions in GC responders and non-responders

Total patients (*n* = 97)	Mutation carriers (*n* = 7)	Mutation non-carriers (*n* = 90)	*P* value
GC responders (*n* = 62)	6 (85.7%)	56 (62.2%)	0.416
GC non-responders (*n* = 35)	1 (14.3%)	34 (37.8%)	

**Fig. 1:**
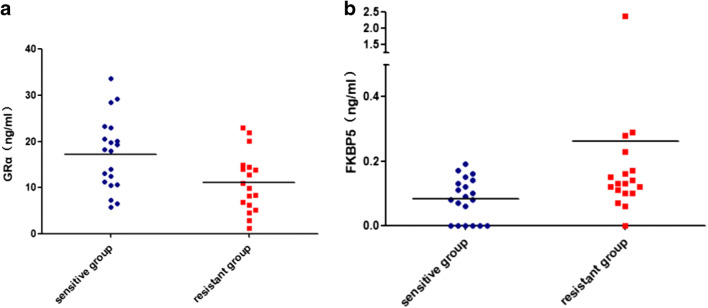
GRα (**a**) and FKBP5 (**b**) serum concentration before GC impulse

ELISA was performed to detect the serum GRα and FKBP5 levels before the GC impulse in patients with different GC sensitivities. The GRα content in the serum of the sensitive group was significantly higher than that of the insensitive group (*P* = 0.011, Fig. 1a). The content of FKBP5 in the sensitive group was significantly lower than the one in the resistant group (*P* = 0.025, Fig. 1b). This result indicated that the high expression of GRα and the low expression of FKBP5 might be related to the increased sensitivity of GC.

## Discussion

This study was designed to explore evidence for the possible correlation of *GR* gene alterations with response to GC. This is the first sequencing of entire *GR* coding exons in Chinese MS patients. We detected seven unreported mutations, whereas the presence or absence of mutations was independent of GC sensitivity. However, previously reported *GR* polymorphisms and mutations related to GC sensitivity were not found [14–17]. To further elucidate the influencing factors of GR sensitivity, we compared the serum GRα and FKBP5 concentrations of GC-sensitive and GC-resistant groups. Our findings showed that the GC-sensitive patients had higher serum GRα and lower FKBP5 levels.

GC is the first-line therapy in acute attack of MS, and GR plays a critical role in the therapeutic effects of GC. Several *GR* gene polymorphisms have been reported to be associated with either glucocorticoid hypersensitivity or glucocorticoid resistance (including *BclI*, *N363S*, *ER22/23EK*, *9β*) in various diseases [16]. For example, van Winsen et al. established that *ER22/23EK* polymorphism was associated with a more aggressive MS phenotype, determined both clinically and on MRI [18]. A study on Guillain-Barre syndrome found that the disease in *BclI* and *Tth111I* carriers was more severe, whereas the *BclI* carriers only had a better prognosis [19]. However, none of the above polymorphisms was detected in any of our 97 MS patients. A possible reason for that outcome is the considerable variations in the frequency of the genotype among various races. The frequency of the G/A genotype of the *ER22/23EK* polymorphism was 0.5% in a healthy Brazilian population and 0% in Asian and African subpopulations [20]. The absence of these polymorphisms in our results could be attributed to ethnic differences and the small sample size.

Many factors influence glucocorticoid sensitivity, including GC bioavailability, GR (gene polymorphisms, splice variant expression, transcriptional activity, and posttranslational modification), and the levels of chaperones and co-chaperones [6]. The *GR* gene is merely one of the factors that influence glucocorticoid sensitivity. On the other hand, FKBP5, as one of the most important co-chaperones, can influence the ligand binding, resulting in changes in the clinical response to glucocorticoid treatment. The degree of dexamethasone-induced expression of FKBP51 in PBMC served as a marker for the clinical response to glucocorticoids in patients with asthma or rheumatoid arthritis [21, 22]. Currently, investigations are undergoing on the application of FKBP51 in drug development [23–25]. Inhibitor of FKBP5 may enhance GC sensitivity and serve as a new drug for GC-resistant patients with acute MS attack.

One of the limitations of this study was the incomplete number of serum samples subjected to ELISA assay; only 38 serum samples (out of 97 cases) were analyzed. Another important limitation was the fact that frozen serum samples rather than PBMCs were available for this retrospective study. Therefore, we failed to perform flow cytometer assay of GRα and FKBP5 on the cellular level. In addition, the interval from the onset to the GC impulse was variable, which might have led to bias in the GC response and efficacy. Finally, EDSS changes were hardly detectable at higher values (> 3); MS functional composite (MSFC) or MS quality of life 54(MSQoL-54) [26, 27] may be more sensitive and suggestive. However, MSFC and MSQoL-54 were unavailable in our study.

In conclusion, the *GR* mutations detected in our study were not associated with the response to GC in Chinese MS patients. The GC-sensitive patients had higher GRα but lower FKBP5 levels, which indicates inhibiting FKBP5 may improve GC efficacy for GC-resistant patients. Based on the findings of our study, we suggest that multiple complicated factors appear to be involved in the GC sensitivity regulation, which warrants further research to develop new drugs for MS.

## Electronic supplementary material

ESM 1 (DOCX 15.3 kb)

ESM 2 (DOCX 987 kb)
