# Supplementary material for: Glucocorticoid receptor mutations and clinical sensitivity to glucocorticoid in Chinese multiple sclerosis patients
Source: Neurol Sci. 2020 Apr 10;41(10):2767–71. doi: 10.1007/s10072-020-04376-8 (PMC7478945; doi:10.1007/s10072-020-04376-8)
Supplement: Supplementary file 1 — (DOCX 15.3 kb) [file 10072_2020_4376_MOESM1_ESM.docx]

| Primer | Primer sequence |
| --- | --- |
| NR3C1-Exon2-1-F-690 | TAAAATCCTCACCGTTGGCC |
| NR3C1-Exon2-1-R-690 | GCCTCTCATTTTACCGGACAC |
| NR3C1-Exon2-2-F-659 | AAGGGATGCTGTATTCATGTCA |
| NR3C1-Exon2-2-R-659 | TGGAAGAAAGCATTGCAAACC |
| NR3C1-Exon2-3-F-671 | AGGTGCTTTGGTCTGTGGTA |
| NR3C1-Exon2-3-R-671 | AGCTGCCTCTTACTAATCGGA |
| NR3C1-Exon3-F-644 | CCTGACCAAACTCCCAGACA |
| NR3C1-Exon3-R-644 | GTGAACCAGAACCACACCAT |
| NR3C1-Exon4-F-439 | TGTGTGTAAGAAGAACTGGTGG |
| NR3C1-Exon4-R-439 | CCTGTGGGTGTCTTGGTTAT |
| NR3C1-Exon5-F-621 | ACCTGTATTCACCTGACTCTCC |
| NR3C1-Exon5-R-621 | CCTCTGGATCTTTTGCTTTCAGA |
| NR3C1-Exon6-F-364 | CAGTCCTGAATTATCACAATAGCA |
| NR3C1-Exon6-R-364 | CCATTTTCTGTTAGGGGTGCC |
| NR3C1-Exon7-F-531 | CCTCTCTGTTTCTGCCATACC |
| NR3C1-Exon7-R-531 | CAGCCAAGATGCAGGAAGTT |
| NR3C1-Exon8-F-480 | TCACCAACATCCACAAACTG |
| NR3C1-Exon8-R-480 | TGCCTACAGTCCTAGCTACT |
| NR3C1-Exon9-F-574 | ACTGCTTCTGTTGCCAAGTC |
| NR3C1-Exon9-R-574 | TGCACCCTTCATATATTATGGT |
