# Supplementary figures and images for: Glucocorticoid receptor mutations and clinical sensitivity to glucocorticoid in Chinese multiple sclerosis patients
Source: Neurol Sci. 2020 Apr 10;41(10):2767–71. doi: 10.1007/s10072-020-04376-8 (PMC7478945; doi:10.1007/s10072-020-04376-8)

Patient No.1


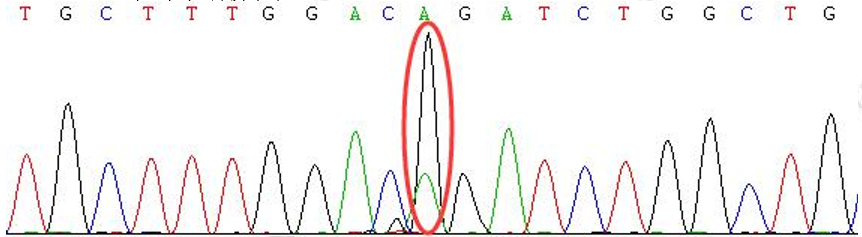


Patient No.2


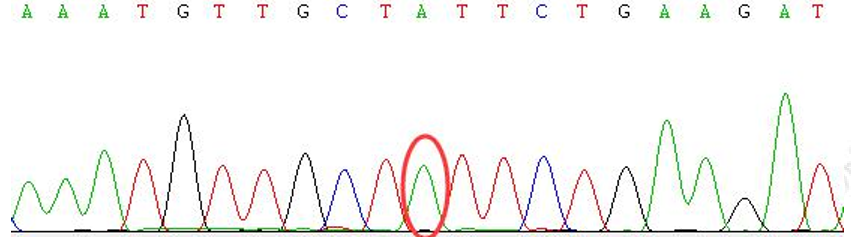


Patient No.3


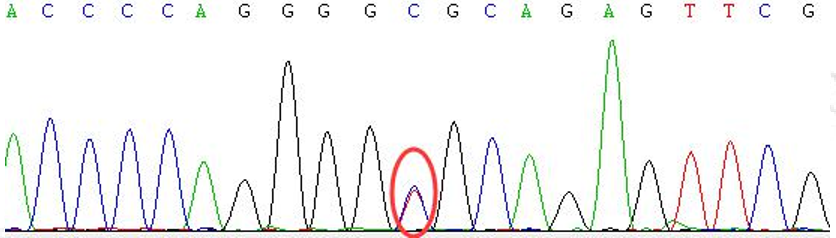


Patient No.4


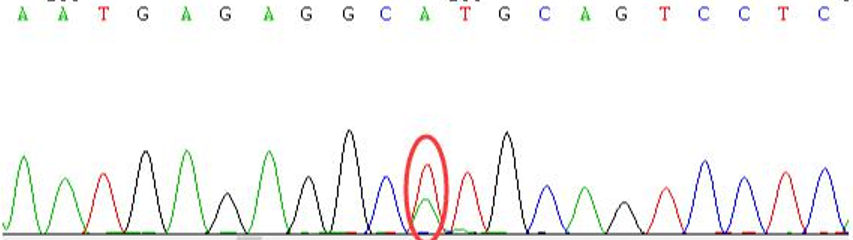


Patient No.5


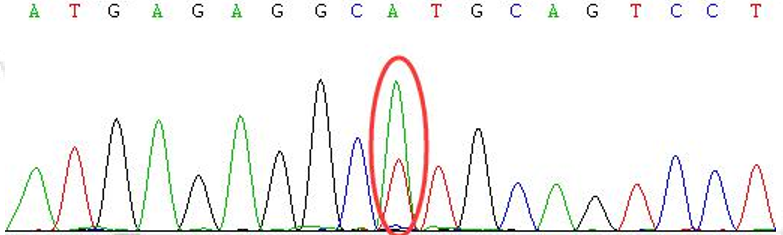


Patient No.6


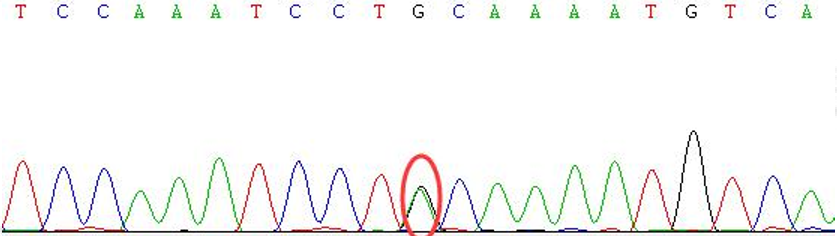


Patient No.7


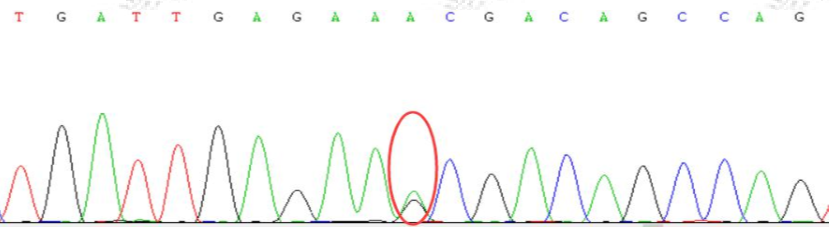

Supplement: Supplementary file 2 — (DOCX 987 kb) [file 10072_2020_4376_MOESM2_ESM.docx]
